# Niche Partitioning in Theropod Dinosaurs: Diet and Habitat Preference in Predators from the Uppermost Cedar Mountain Formation (Utah, U.S.A.)

**DOI:** 10.1038/s41598-018-35689-6

**Published:** 2018-12-14

**Authors:** J. A. Frederickson, M. H. Engel, R. L. Cifelli

**Affiliations:** 1 Department of Biological Sciences, Southwestern Oklahoma State University, 100 Campus Drive, Weatherford, OK 73096 USA; 2 Department of Biology, University of Oklahoma, Norman, Oklahoma 73072 USA; 3 School of Geology and Geophysics, University of Oklahoma, Norman, Oklahoma 73072 USA; 4 Sam Noble Museum, 2401 Chautauqua Avenue, Norman, Oklahoma 73072 USA

## Abstract

We explore hypothetical ecologies to explain diversity among predatory dinosaurs in North America’s medial Cretaceous, based on occurrence, tooth morphology, and stable isotope analysis. The Mussentuchit local fauna, Utah, USA, is among the best-known terrestrial vertebrate assemblages from the Cretaceous. Study samples include teeth from six microvertebrate sites, ranging in depositional setting from distal floodplain to channel lags. We recognize four theropod morphotypes: a comparatively large theropod (morph 1), a medium-sized dromaeosaurid (morph 2), a small dromaeosaurid (morph 3), and a tooth-morph similar to the genus *Richardoestesia* (morph 4). These four morphotypes vary significantly in mean size, from 15.1 mm in the largest theropod to 3.7 mm in *Richardoestesia*. Further, tooth representation from two of the best-sampled microsites (representing a channel/splay and floodplain deposit) show differing patterns of abundances with morphs 1 and 3 having roughly the same abundance in both sites, while morph 2 was more abundant in the floodplain setting and morph 4 was more abundant in the channel/splay. Stable isotope analysis (δ^13^C; δ^18^O) of tooth carbonate from the theropod morphotypes, goniopholidid crocodilians, and matrix (to test for diagenesis) from these sites were also analyzed. The theropods show modest differences in δ^13^C values between each other, with carbonate from the teeth of morphs 1, 3, and 4 being enriched in ^13^C for the channel/splay relative to the floodplain environments, possibly indicative of dietary plasticity in these species. We hypothesize that these data indicate that the Mussentuchit theropods had different niches within the predator guild, suggesting plausible means by which ecospace was divided among the predatory dinosaurs of the Mussentuchit local fauna.

## Introduction

Niche partitioning is an ecological phenomenon wherein multiple competing organisms coexist in the same environment by maximizing their occupation of non-overlapping lifestyles. Although generally well documented in modern species, demonstrating niche partitioning in Mesozoic ecosystems has been challenging. Much of this difficulty has been alleviated through decades of research showing that dinosaurs were highly derived animals with complex ecological relationships (e.g.^1^). In no group has this realization been more apparent than in non-avian theropods, which possess many morphological specializations associated with unique lifestyles, such as pachyostotic bones and dorsally-placed nostrils in aquatic spinosaurids^2^, digging-style claws in alvarezsaurids^3^, and edentulous beaks in multiple herbivorous clades^4^; all of which likely evolved to exploit food sources unavailable to other theropods. In addition, there is evidence that some members of this group were capable of complex behaviors, such as pack hunting^5^, which would further differentiate their predatory trophic abilities. Even so, theropod ecology, in particular relationships between coexisting (sympatric) species, is poorly understood. A major impasse has been the facts that most theropods (besides those mentioned above) do not possess obvious adaptations associated with particular diets, and that many taxa lack representation by anatomically informative fossils. Without further lines of evidence, determining the precise trophic ecology of these dinosaurs is problematic beyond characterization as “generalist predator”, a label that could reasonably be attributed to any sharp-toothed species.

In all well-sampled Late Cretaceous terrestrial faunas, multiple small- to medium-sized theropods coexisted^6^. These faunas include: dromaeosaurs, troodontids, and small (or juvenile) tyrannosaurs, each possessing clear anatomical specialization for predatory lifestyles. Adaptations, such as sharp, recurved teeth and long sickle-shaped claws with enlarged basal tubercles (for attachment of flexor tendons), broadly suggest that these theropods would be in direct competition if they preyed upon the same species. This high diversity of competitors seemingly contradicts the Competitive Exclusion Principal^7^, which states that species occupying the same niche cannot exist indefinitely in the same environment. In order to maintain such high diversity, these predatory species would have had to minimize competition amongst themselves through behavioral or spatial dietary segregation. Modern animals provide good examples of how niche partitioning can be manifested, primarily by specializing in different prey items (e.g.^8^), feeding at different times of day (e.g.^9^), or occupying different subenvironments (e.g.^10^). By adopting one (or more) of these strategies, similar organisms can coexist without directly competing for resources. Thus, it stands to reason that in diverse theropod communities, we would expect to see some level of niche partitioning following the strategies observed in modern assemblages.

Determining behavior from the fossil record is a difficult task. It can be assumed that animals are adapted to a specific lifestyle, allowing for broad generalizations about the animal’s ecology based on morphology alone. For example, the sharp teeth and long legs of dromaeosaurids leave little doubt that these animals were cursorial predators; however, what they were eating cannot be precisely determined by morphology alone. In the best-case scenario, animals are preserved in the act of feeding or with stomach contents still in place. Famous examples such as the fighting dinosaurs, a *Velociraptor mongoliensis* and *Protoceratops andrewsi* preserved in an eternal predator-prey struggle^11^, unambiguously show that this trophic relationship occurred at least once. Even further, trackways occasionally show evidence of presumed hunting or packing behavior^12^. Fossils of this ecological caliber are rare, leaving paleoecologists to rely on more indirect proxies for ancient behavior; namely, through geochemical or taphonomic evidence. To date, few of these ecological studies have focused on theropods, and when performed most focus on the large or enigmatic species (e.g.^13,14^). Unsurprisingly, it is mainly in these large species that strong evidence for niche partitioning has been presented^15^.

In this study, we investigate the realized dietary niche of theropods from relatively well-sampled micro-bonebeds, derived from the Late Cretaceous Mussentuchit Member (herein referred to as MM) of the Cedar Mountain Formation of Utah. Through morphological, taphonomic, and geochemical techniques we investigated the dietary and habitat preferences of superficially similar species, in order to more fully understand the Mussentuchit ecosystem, as well as the ecology of many of these poorly-known theropods.

## Geologic Setting

**Figure 1:**
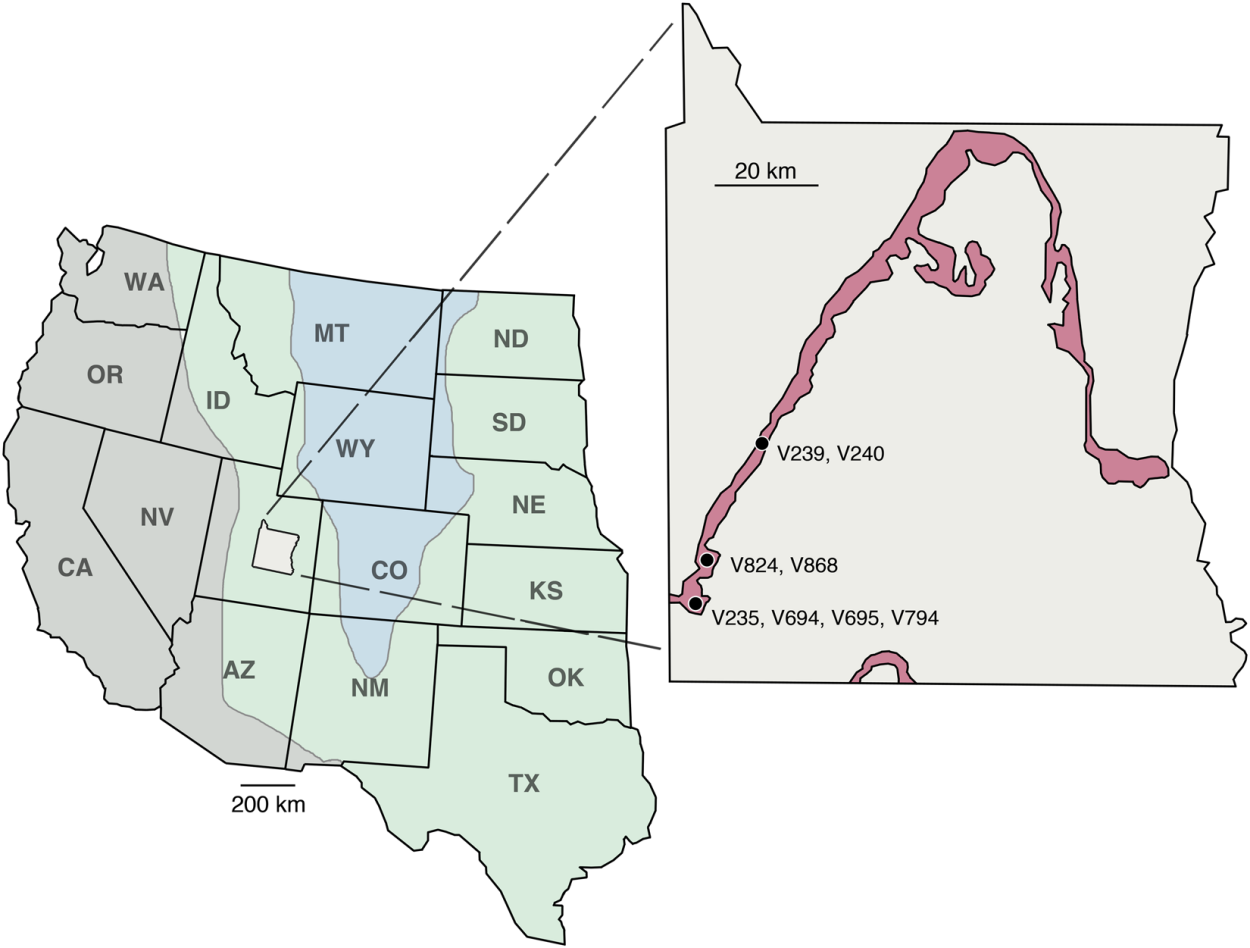
Map of the western interior during the Cenomanian (grey is highlands, green is lowlands, and blue is water) and a pullout of Emery County, Utah with the Mussentuchit Member exposure and microsites analyzed in this study. Based on maps and data from Cifelli *et al*.^21^ and Suarez *et al*.^17^.

The MM is the uppermost unit of the Cedar Mountain Formation exposed on the western side of the San Rafael Swell anticline in Emery County, Utah (Fig. 1). This unit is composed of terrestrial sediments, varying between sandstones, mudstones, and altered volcanic ash layers; the latter of which comprise much of the grey, smectite-rich badlands characteristic of the unit^16,17^. The MM formed as shed sediments deposited in the foreland basin during the Pavast thrust event, dated from 98.3 ± 0.1 Ma^18^ to 96.7 ± 0.5 Ma^19^, placing the member in the Cenomanian stage of the Late Cretaceous. The unit is generally highly fossiliferous (as compared to the rest of the Cedar Mountain Formation), with a diverse fauna composed of freshwater fishes, lissamphibians, lepidosaurs, crocodilians, dinosaurs, and mammals^19,20^.

The samples analyzed here come from six micro-bonebeds collected by crews from the Sam Noble Museum in the Mussentuchit Wash and Short Canyon areas of Emery County, Utah^17,21,22^. Stratigraphically, localities V235, V694, and V794 are located near the same level, above a marker ash bed, 15 m from the base of the overlying Naturita Formation (formerly referred to as the Dakota Formation in the region^23^), while V695 is found immediately below this same layer. V868 and V239 are found nearer to the contact of the MM with the overlying Naturita Formation. Though they differ in depositional setting (see Table 1), Goldberg^22^ showed that faunal composition between the sites was not dramatically affected by differences in taphonomic history. Further, specimens between sites tend to show a similar minor degree of wear attributable to transportation, indicating that hydraulic transport and reworking was minimal. Most importantly for our purposes, previous isotopic analyses from this same sample demonstrate the expected variation for modern fauna, implying that biogenic signals were not lost due to diagenetic alteration^17,24^.

**Table 1:** Depositional environments used in this study. C = channel, C/S = channel/splay, S/F = splay/floodplain, F = floodplain. Data from Goldberg^22^ and Suarez *et al*.^17^.

Microsite	868	794	695	694	239	235
Dep. Env.	C/S	C/S	F	F	C	S/F
m below Naturita Fm	8	15	19	15	4	15

## Descriptions

### Tooth morphotypes

In total, 866 small- to medium-sized theropod teeth from the MM were analyzed and found to represent at least seven different morphotypes, many of which have been identified elsewhere^19,21,25^. Here we recognize the following morphotypes in our sample sites:

#### Morphotype 1

**Figure 2:**
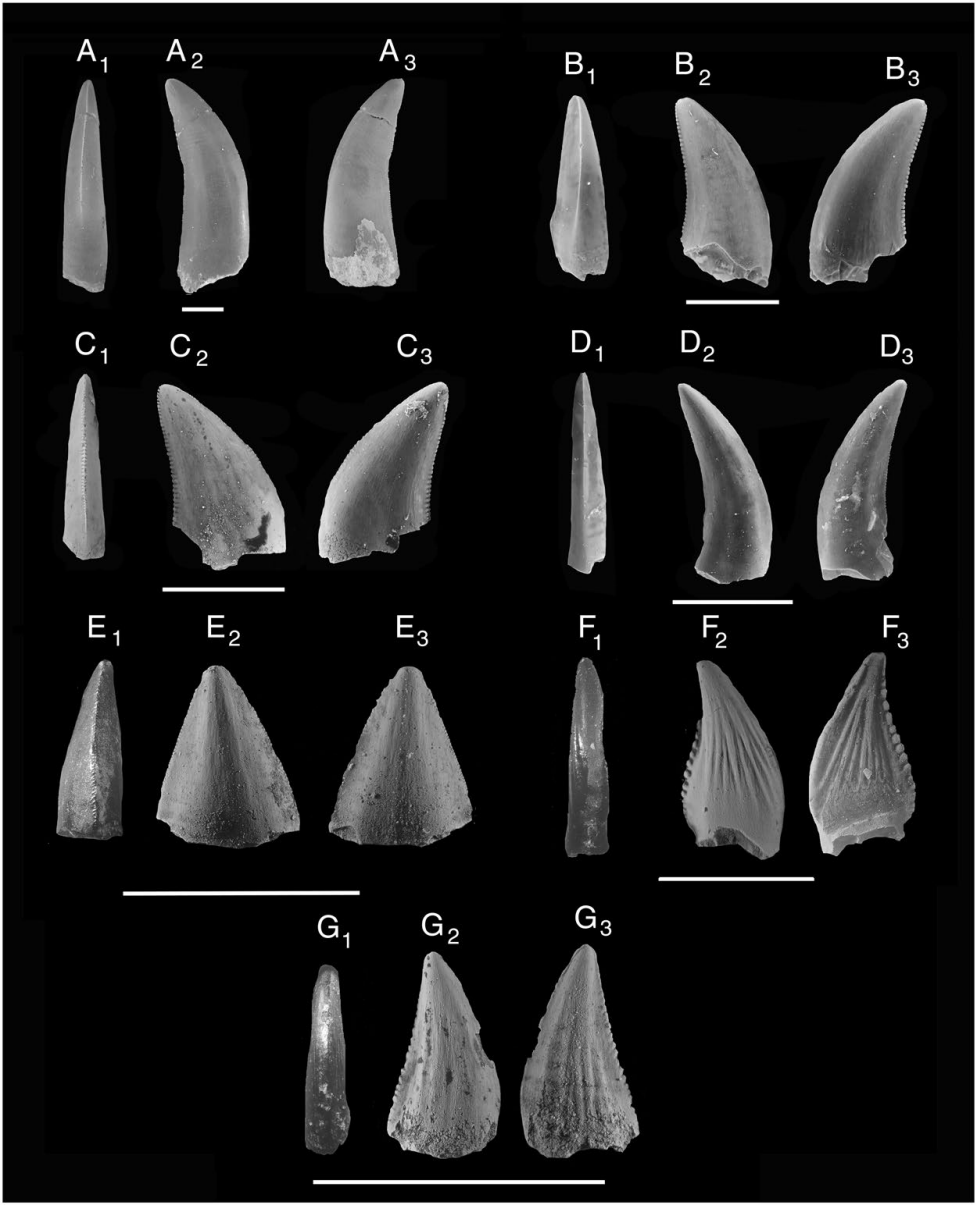
Tooth morphotypes from the Mussentuchit Member. (A_1–3_) Morph 1, largest theropod; (B_1–3_) morph 2, medium-sized dromaeosaurid; (C_1–3_) morph 3, small dromaeosaurid; (D_1–3_) morph 4, *Richardoestesia* indet.; (E_1–3_) morph 5, cf. *Richardoestesia isosceles*; (F1–3) Morph 6, *Paronychodon* indet.; (G_1–3_) morph 7,?troodontid. Scale is 5 mm.

These teeth are the tallest of the morphotypes analyzed, with the largest being approximately 35 mm in crown height (Fig. 2A_1–3_). These teeth are not as laterally compressed as other morphotypes and possess denticles on both carinae. The denticles on the anterior carina are proximodistally short, apicodistally wide, and oval in cross-section; the denticles on the posterior carina are proximodistally long and chisel-shaped. These teeth are likely the same as “Theropod A” of Fiorillo^25^. The teeth are of uncertain origin, showing similarities with tyrannosaurs (previously reported from the unit by Cifelli *et al*.^21^), but lack the basally-deflected blood groove diagnostic of this group^26^. These teeth are similar in size and appearance to those described as morphotype 1 by Krumenacker *et al*.^27^ from the contemporaneous Wayan Formation of Idaho. Lacking more complete material, these authors tentatively refer these teeth to a moderately-sized tyrannosauroid or basal tetanuran. The only large-bodied theropod currently known from the MM is *Siats meekerorum*^28^; unfortunately, the holotype (and only definitively known specimen) contains no teeth. Regardless, among the huge number of theropod teeth recovered from the MM, none is large enough to belong to an adult *S. meekerorum*.

#### Morphotype 2

Reaching heights up to 15.69 mm tall, these teeth are generally shorter than morphotype 1, but taller than the other morphotypes (Fig. 2B_1–3_). The lingual side is often flattened, while the labial side is more inflated, forming a slight D in cross section. The anterior carina rarely possesses denticles, and curves lingually as it travels towards the base, a feature seen most prominently in lateral teeth of *Dromaeosaurus*, as well as anterior teeth of other dromaeosaurids^29^. The posterior denticles are relatively elongate and rounded on the ends. These teeth match the description for Dromaeosaurinae teeth by Fiorillo^25^, and those figured by Garrison *et al*.^19^.

#### Morphotype 3

Morphotype 3 is composed of teeth that are generally small and relatively recurved as compared to morphotypes 1 and 2 (Fig. 2C_1–3_). These teeth possess denticles on both carinae, with the anterior denticles being wide, low, and rounded; while the posterior denticles are taller, and point slightly apically. Unlike morphotype 2, the anterior carina never curves lingually. The large, upturned posterior denticles with subequal-sized anterior denticles identify these teeth as belonging to a small species of dromaeosaurid^6^. Morphologically similar, but relatively larger teeth are also known from the contemporaneous Wayan Formation of Idaho^27^.

#### Morphotype 4

**Figure 3:**
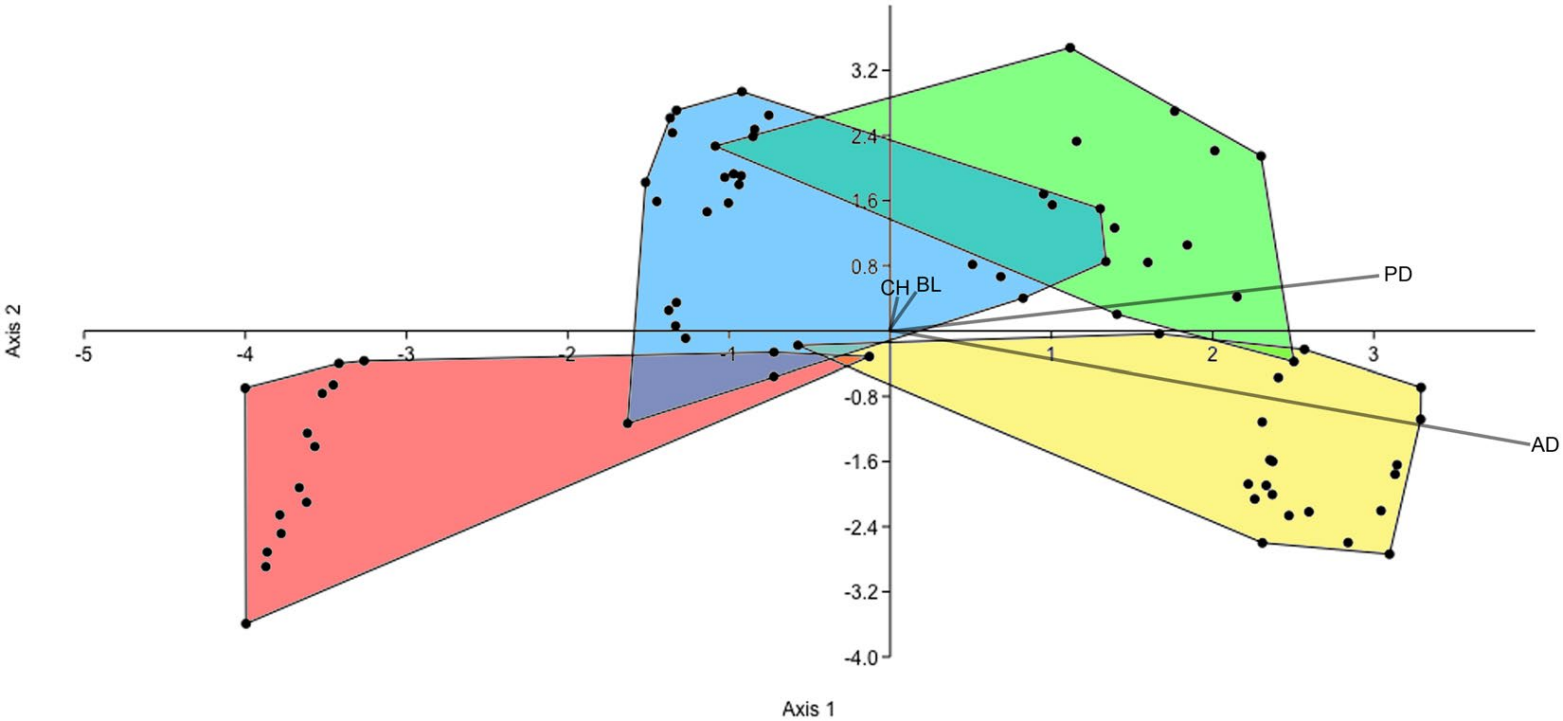
Discriminant analysis for the four most distinct, best-sampled morphotypes (morph 1 is blue, morph 2 is green, morph 3 is yellow, and morph 4 is red). Axis 1 and 2 account for 93.72% of the maximum discrimination. CH is crown height, BL is basal length, PD is posterior denticle count, and AD is anterior denticle count.

Relatively small, tall, and relatively rounded, these teeth are only moderately recurved (Fig. 2D_1–3_). In most specimens denticles are absent from both carinae; when present, they are relatively small and low and usually only found on the posterior end. In the largest specimens, there are 9–10 denticles per mm; however, most lack them completely. Some of these specimens show a slight lingual curvature on the anterior carina, indicating that these may be anteriorly-located teeth. These teeth are nearly identical to those identified as tall variations of cf. *Richardoestesia isosceles* from the Santonian Milk River Formation of Canada^30^ (Fig. 3G). Due in part to substantial variation known for *Richardoestesia* teeth^26^, we cannot confidently assign these teeth beyond the generic level.

#### Morphotype 5

Triangular teeth with relatively small denticles on both carinae (9–10 per mm) were first identified in the MM by Cifelli *et al*.^21^ and subsequently described by Garrison *et al*.^19^ (Fig. 2E_1–3_). These teeth match most closely those described as cf. *Richardoestesia* sp., though their small, rounded denticles and isosceles-triangular shape in lateral view are unquestionably similar to those of the enigmatic, and possibly non-theropodan^31^, *Richardoestesia isosceles* from the Late Cretaceous Aguja Formation of Texas^32^. Similar triangular teeth are known from the contemporaneous Woodbine Formation of Texas, which have also been attributed to *Richardoestesia*^33^. It is possible that morphs 4 and 5 represent teeth from a single species, but without any associated material this is simply conjecture.

#### Morphotype 6

These relatively tall teeth with one flattened side and longitudinal grooves are similar to *Paronychodon* specimens described from other Late Cretaceous formations (Fig. 2F_1–3_). Conversely, the *Paronychodon* specimens from the MM typically have large, rounded denticles on the posterior carina, while those in geologically younger formations often lack denticles entirely^6^. Currie *et al*.^26^ hypothesized that these teeth do not belong to a unique taxon, but instead represent malformed dromaeosaur teeth. Without more complete material it is difficult for us to contribute to this discussion; however, the general scarcity of morph 6 to other theropod teeth supports the assertion that these teeth are malformations.

#### Morphotype 7

Slightly recurved teeth, lacking denticles on the anterior carina and possessing apically-upturned denticles on the posterior carina (Fig. 2F_1–3_) are most similar to those identified from the MM as Velociraptorinae by Fiorillo^25^. These teeth are often very short (less than 5 mm long), with the smallest containing as few as seven denticles. These diminutive specimens superficially appear most similar to *Troodon* teeth and were originally identified as such by Cifelli *et al*.^21^ and Goldberg^22^; however, morphologically similar, but larger, teeth retain the same number of denticles per mm (5–6), implying that they belong to the same species differing only in ontogenetic status or alveolar position. These larger teeth have relatively smaller denticles than those of most Late Cretaceous troodontids (e.g. Fig. 2H of Larson and Currie^6^); though more associated material is needed to unequivocally dismiss this identification. Some specimens appear to bear longitudinal grooves, similar to those of Morph 6, but to a lesser degree. These individuals may also belong to the same species as Morph 6, which themselves may belong to one of the other morphotypes.

Though the MM theropods are a seemingly diverse assemblage, it is likely that multiple morphotypes belong to a single species. For the remainder of this study we focused on four of the most common and morphologically discrete morphotypes (morphs 1–4; 309 specimens of the subsample) to maximize the likelihood that we analyzed distinct species. The taxonomic identifications are tentative, based on the lack of more complete material from the MM. Regardless, these identifications are peripheral to our main concern, the relative differences in distribution and inferred diet.

## Results and Discussion

### Ordination

A discriminant analysis of the four morphotypes conducted on 77 complete teeth found that 83.12% of all teeth were correctly identified. In this analysis, axes 1 and 2 account for 61.5% and 32.22% of the maximum discrimination, respectively; and the biplot shows the strongest contributions from the anterior and posterior denticle counts largely in the direction of axis 2 (Fig. 3). Morph 4 was the most consistently identified morph, with 93.3% of specimens correctly placed and only one specimen misidentified as belonging to morph 2. Morph 3 was identified correctly 90.9% of the time, with two out of the 22 specimens misidentified as belonging to morph 1. Morph 2 was identified 80.8% of the time correctly, with morph 4 being the most commonly mistaken morph (11.5%) and morphs 3 and 1 each predicted once (3.8%). Morph 1 was the most poorly-predicted with only a 64.3% success rate, where the other five out of the 14 specimens are identified as morph 2. Following Larson and Currie^6^, hit ratios between 75–100% can be considered quantitatively distinct morphs, as opposed to Hammer and Harper^34^ who limit this threshold to 90% and above. Using either guideline, morphs 3 and 4 can be differentiated based on these measurements alone, while morph 2 can only be recognized based on the more liberal categorization. In neither case is the analysis sufficient to identify morph 1 using measurements alone. However, based on its inflated widths, denticle shape, and lack of a lingually-curved posterior carina, it is sufficiently likely that morph 1 can be recognized based on qualitative characteristics. We tentatively accept the overall hit-ratio as indicative that all four morphs are sufficiently different to consider them as separate taxonomic entities.

### Size and inferred diet

**Figure 4:**
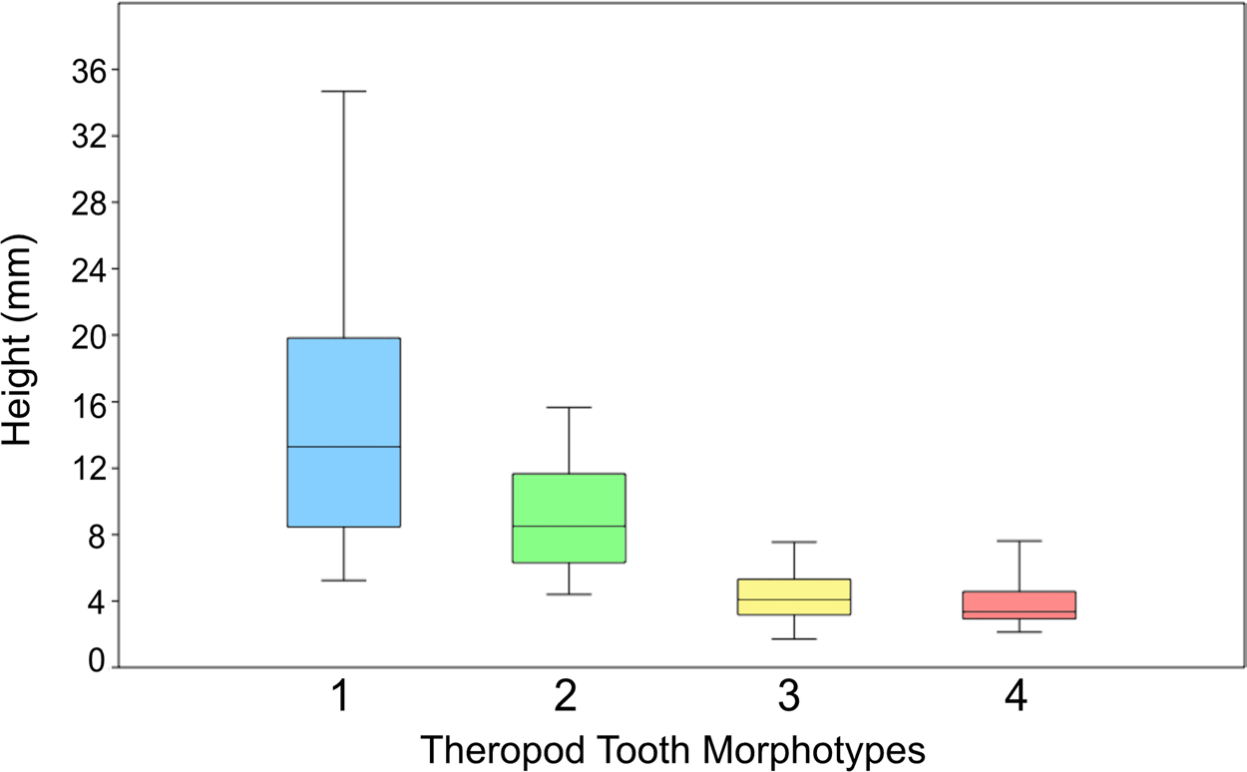
Size (in mm) of crown height for each tooth morphotype (morph 1 n = 15; morph 2 n = 35; morph 3 n = 69; morph 4 n = 39). Color scheme follows Fig. 3.

The four morphotypes vary significantly in mean size, from 15.1 mm in morph 1 (5.2–34.7 mm) to 3.7 mm in morph 4 (2.1–7.6 mm), arranged into multiple size classes (Fig. 4). The results of a Kruskal-Wallis test show that each of the four morphs have unequal medians (p < 0.01), indicating that regardless of overlap between the smallest and largest teeth of any two morphs there is a distinct difference in size among the four species. This size diversity would presumably translate into differential trophic abilities for each morph.

The largest teeth from morph 1 are comparable in size to those of gracile tyrannosaurids from the Late Cretaceous, such as the 4 to 5 m-long *Alioramus altai*^35^. Hypothetically, this morph would have been capable of feeding on varying-sized prey, including the other, smaller morphotypes. Morph 2 has the next largest teeth, with crown heights (up to 15.7 mm tall) often exceeding those of *Deinonychus antirrhopus*, a 3 m-long dromaeosaurid from the Aptian/Albian of North America^36^, which possesses teeth rarely surpassing 15 mm in height^37^. Strong evidence exists that *Deinonychus antirrhopus* habitually fed upon the iguanodontian *Tenontosaurus tilletti*^5,38^. This trophic relationship led to the widely-touted hypothesis that, like modern canids, *Deinonychus* could have used pack hunting strategies to catch and dispatch larger prey^39^; although this hypothesis has recently been challenged (see^40^ for further discussion). Morph 2’s superior size, as well as the possibility of pack hunting behavior, indicates that this species would have been a relatively uninhibited predator, though like *Deinonychus* it may have specialized in hunting ornithopod dinosaurs (such as the ubiquitous *Eolambia caroljonesa*). Morph 3, unlike morph 1 or 2, would have faced more dietary limitations due to its small size. This small dromaeosaurid has teeth ranging from 1.7 to 7.6 mm tall, roughly equivalent to teeth from the 1 to 2 m-long Late Cretaceous *Bambiraptor feinbergi*^41^. This small size likely limited this species to feeding upon smaller prey, such as baby dinosaurs, mammals, reptiles, amphibians, and fish. Even smaller still is morph 4, which we tentatively assigned to the genus *Richardoestesia*. This genus was originally named for lower jaws and numerous isolated teeth discovered in Campanian-age sediments of Western Canada^26^. *Richardoestesia* teeth are thought to belong to a piscivorous species, based on its elongate dentary, high tooth count, and straight crowns with reduced curvature and minute denticles^26,29^. Further, apical wear patterns on *Richardoestesia* teeth from the latest Cretaceous are consistent with a fish-eating diet^29^. Given what is known about this genus, it is possible that morph 4 had a diet similar to modern wading birds (such as ardeids), consisting of fish as well as opportunistically feeding upon small mammals, reptiles, amphibians, and invertebrates^42,43^.

### Taphonomy

**Figure 5:**
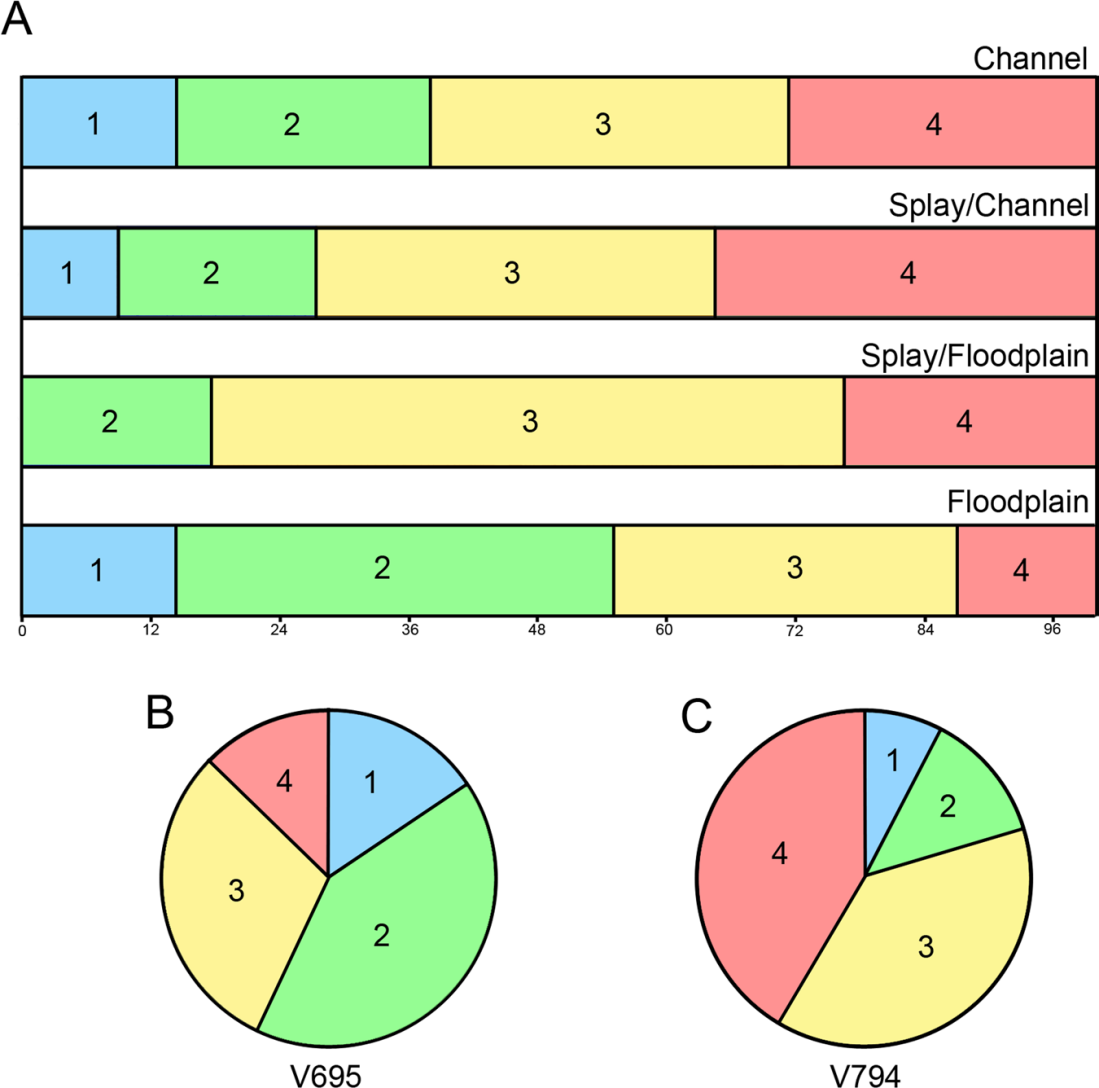
(**A**) percent composition for each morphotype in deposits identified as channel (n = 21, V239), splay/channel (n = 124, V794 and V868), splay/floodplain (n = 17, V235), and floodplain (n = 147, V694 and V695); (**B**) pie chart showing the tooth composition of V695 (n = 135; floodplain); (**C**) pie chart showing the tooth composition of V794 (n = 104; splay/channel). Color scheme follows Fig. 3.

Among the four depositional environments represented across the six microsites (Table 1), there are distinct differences in the relative abundances of the morphotypes. Unfortunately, small sample sizes at many of the microsites prevent a robust characterization of their respective assemblages. To address this limitation, sites were grouped and analyzed based on their inferred depositional setting (Table 1; Fig. 5A). In the grouped data sets, specimens belonging to morph 1 (largest theropod) are found in lower abundances than the other three morphotypes, making up approximately 14.3% of the teeth across the environment (channel and floodplain settings), and never accounting for more than 16% from any single microsite. Conversely, teeth assigned to morph 3 (small dromaeosaur) are the most consistently abundant, composing between 31.9% (in floodplains) to 58.8% (in splay/floodplains) of the observed population. Unlike the previous two examples, fossils belonging to morph 2 (medium dromaeosaur) are unequally distributed, and show a general trend of increasing in relative abundance moving distally from the channels. This morph only makes up 23.8% of channel deposit abundance, while composing 40.8% of the floodplains census. In direct contrast, morph 4 (*Richardoestesia* sp.) shows an increased relative abundance moving from the floodplains (12.9%) to the channels (28.6%), with the largest abundance in the splay/channel environments (35.4%). When constrained to only microsites with substantial sample sizes (Fig. 5B,C), morph 1 is about twice as common in the floodplain environment (V695; 15.6%) compared to the splay/channel (V794; 7.7%); while morph 2 teeth are more than three times as common in the floodplain (12.5% to 41.5% respectively). Morph 3 show little difference between the two sites (30.4% to 38.5%), and Morph 4 increase more than three-fold from the floodplain to the splay/channel (12.6% to 41.3%). These differences are significantly non-random (χ^2^ = 40.57, d.f. = 3, p < 0.01). Morph 1 and morph 3 are not skewed toward either depositional environment (χ^2^ = 2.93, p = 0.40; χ^2^ = 0.89, p = 0.82), while morph 2 are statistically more abundant in the floodplain (V695; χ^2^ = 25.88, p < 0.01) than morph 4, which are more abundant in the splay/channel (V794; χ^2^ = 22.05, p < 0.01).

Initial comparisons between the two microsites (V794 and V695) would seemingly indicate a bias based on size, possibly due to hydrologic sorting. To test this possibility, we looked at the distribution of teeth for the much more abundant goniopholidid crocodilians from both sites. In general, V695 (n = 222) has the tallest teeth (range from 0.85 to 30.43 mm, mean = 6.16 mm, median = 4.36 mm), while V794 (n = 184) has the smallest (0.65 to 22.45 mm, mean = 6.08 mm, median = 5.65 mm). However, both sites show a strong bias toward the smallest teeth and neither site was found to be significantly different using a two-tailed Mann-Whitney U-test (U = 18600, p = 0.12). In contrast, the theropod data, when pooled, show a significant difference in size, where V695 (n = 68) contains significantly taller teeth than V794 (n = 53) (U = 869, p < 0.01). This result, however, is unsurprising given the higher abundance of the largest theropod (morph 1) teeth at V695 relative to V794. To test for size differences within theropod groups, we performed the same analysis for morph 3 (n = 25), the most abundant theropod at both sites. As in the goniopholidids, morph 3 shows a higher mean tooth height in V695 (4.50 mm; median = 4.36 mm) compared to V794 (4.07 mm; median = 3.78 mm); however, a Mann-Whitney U-test showed no significant differences between these populations (U = 244.5; p = 0.19). These distributions suggest that hydrologic sorting is an unlikely explanation for the distribution of theropod morphs among the site samples (see also Goldberg^22^).

An alternative hypothesis is that the distribution of theropods in these sites is controlled by stratigraphy, not by the environments in which these sites formed. Although all four morphotypes are found in the stratigraphically lowest (V868 and V695) and highest (V239) sites, the distribution may not be consistent. Indeed, relative abundances at V868 are more similar to those of V695, a site at the stratigraphically similar position, than to V794, a site reflecting the same depositional environment^22^. Conversely, the stratigraphically equivalent V694, V235, and V794 vary in depositional setting, and show few similarities in their respective morphotype abundances, particularly with respect to morph 2 and morph 4. Given the small sample sizes from these sites (excluding V794 and V695) and their limited vertical distribution, stratigraphic occurrence insufficiently explains theropod distribution and abundance among the sites. Instead, we hypothesize that, at least in part, the distribution of the MM theropod morphotypes is a result of behavior by the organisms causing preferential burial in certain environments. Habitat preference, as inferred from depositional setting, is consistent with the morphology of respective tooth types, such as an affinity for aquatic parts of the fluvial system in the hypothetically piscivorous *Richardoestesia* teeth (morph 4) and a penchant for floodplain settings in the proto-typical theropod teeth of the medium dromaeosaurid (morph 2). These findings also generally agree with predictions based on modern ecosystems^44^; such as the scarcity of larger predators (morph 1) when compared with smaller species (such as morph 3).

### Geochemical analysis

**Figure 6:**
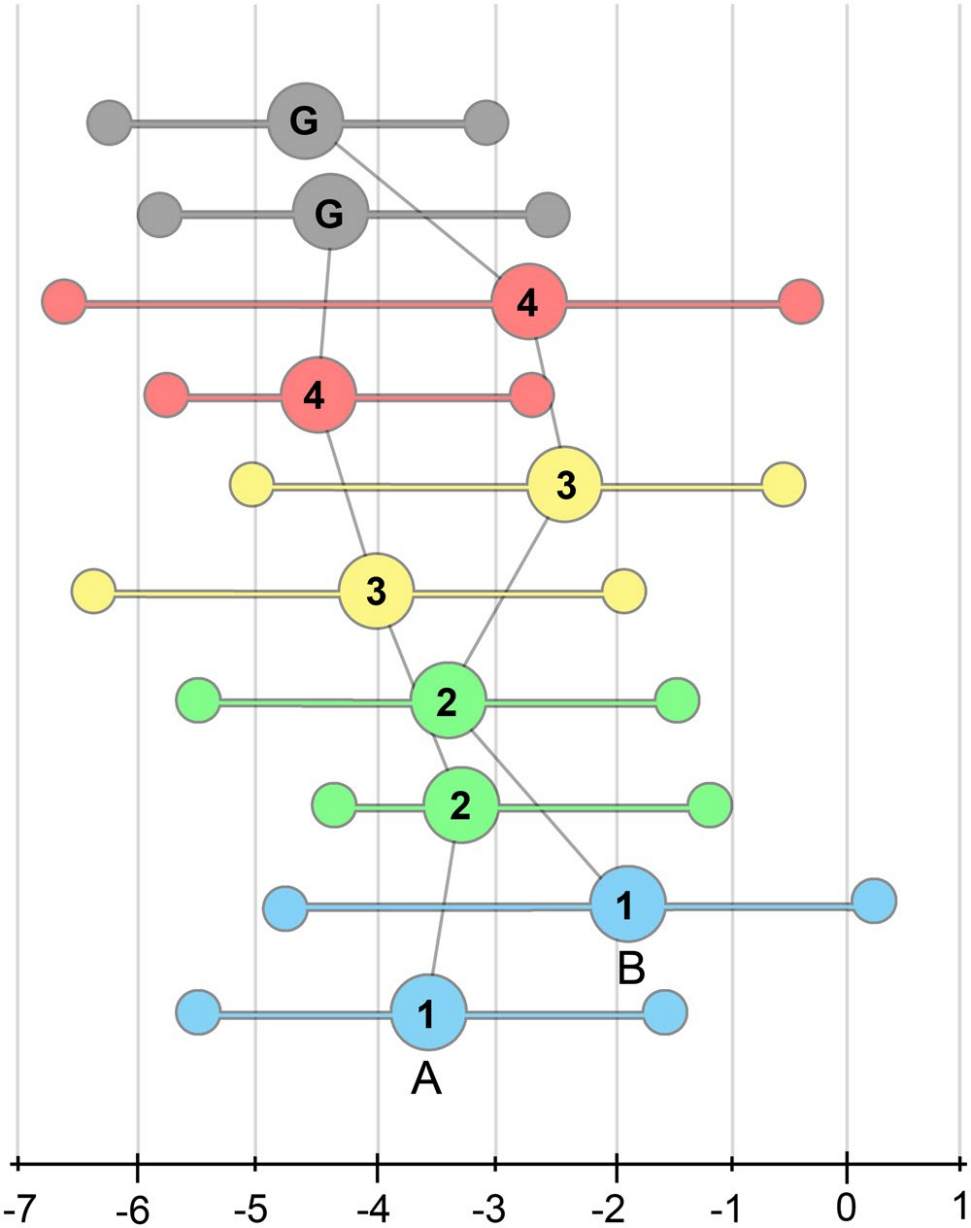
δ^13^C max, min, and mean for four theropod morphotypes (1 through 4) and goniopholidids (G) in (**A**) V695 and (**B**) V794. Morphs 1, 3, and 4 show a significant increase in δ^13^C between V695 and V794, while morph 2 and goniopholidids show no variation. Color scheme follows Fig. 3.

Stable isotope analyses of tooth-associated carbonate (δ^13^C and δ^18^O) were conducted on all four morphotypes for both V695 and V794 (the largest microsites representing both a floodplain and channel/splay environment), as well as samples of small goniopholidid crocodilian teeth and rock matrix. The δ^18^O values showed no statistical difference between morphotypes, but did differ between the matrix and all morphotypes at V794 (p < 0.05) (Table 2). However, differences in δ^13^C values between morphotypes are somewhat more significant (Table 3; Fig. 6). Between the morphotypes in V695, morph 4 (*Richardoestesia* sp.; n = 6, mean δ^13^C = −4.38‰, SD = 1.15‰) and morph 2 (medium dromaeosaur; n = 12, mean δ^13^C = −3.18‰, SD = 1.07‰) showed the largest difference (1.2‰; p = 0.08). Goniopholidids in V695 had mean δ^13^C values similar to morph 4 (n = 10, mean δ^13^C = −4.31‰, SD = 1.09‰), but were depleted in ^13^C relative to morph 3 (small dromaeosaur; n = 10, mean δ^13^C = −3.75‰, SD = 1.41‰) and morph 1 (largest theropod; n = 11, mean δ^13^C = −3.62, SD = 1.29‰) respectively. In V794, goniopholidids retain a relatively low mean average (n = 10, mean δ^13^C = −4.55‰, SD = 0.67‰) that is significantly depleted in ^13^C relative to all of the theropod morphotypes (p < 0.05). Unlike in V695, however, morph 2 (n = 8) has the lowest mean average (n = 8, mean δ^13^C = −3.03‰, SD = 1.52‰), followed by morph 4 (n = 13, mean δ^13^C = −2.72‰, SD = 1.66‰), morph 3 (n = 10, mean δ^13^C = −2.38‰, SD = 1.32‰), and morph 1 (n = 7, mean δ^13^C = −1.93‰, SD = 1.80‰), respectively. In all morphotypes except morph 3, variance increased between the V695 and V794 sites.

**Table 2:** δ^18^O data for all morphotypes, goniopholidid teeth, and matrix from both V695 and V794. C/S = channel/splay and F = floodplain.

Morphotypes	Microsites	N_samples_	Min‰	Max‰	Avg‰ ± 1σ
1	V695 (F)	11	16.35	21.94	20.23 ± 1.56
	V794 (C/S)	7	19.84	21.42	20.83 ± 0.59
2	V695 (F)	12	19.12	21.35	20.46 ± 0.66
	V794 (C/S)	8	18.75	21.73	20.32 ± 0.89
3	V695 (F)	10	18.84	22.66	20.50 ± 0.99
	V794 (C/S)	10	19.29	23.13	21.02 ± 1.13
4	V695 (F)	6	19.61	21.29	20.16 ± 0.58
	V794 (C/S)	13	19.60	21.06	20.55 ± 0.50
Goniopholidid	V695 (F)	10	19.70	21.04	20.53 ± 0.44
	V794 (C/S)	10	19.99	22.54	20.82 ± 0.75
Matrix	V695 (F)	12	18.78	25.31	21.36 ± 2.11
	V794 (C/S)	6	16.6	20.23	17.81 ± 1.28

**Table 3:** δ^13^C data for all morphotypes, goniopholidid teeth, and matrix from both V695 and V794. C/S = channel/splay and F = floodplain.

Morphotypes	Microsites	N_samples_	Min‰	Max‰	Avg‰ ± 1σ
1	V695 (F)	11	−5.49	−1.65	−3.62 ± 1.29
	V794 (C/S)	7	−4.38	0.76	−1.93 ± 1.80
2	V695 (F)	12	−4.42	−1.24	−3.18 ± 1.07
	V794 (C/S)	8	−5.79	−1.38	−3.03 ± 1.52
3	V695 (F)	10	−6.30	−1.89	−3.75 ± 1.41
	V794 (C/S)	10	−4.47	−0.50	−2.38 ± 1.32
4	V695 (F)	6	−5.74	−2.69	−4.38 ± 1.15
	V794 (C/S)	13	−6.44	−0.20	−2.72 ± 1.66
Goniopholidid	V695 (F)	10	−5.99	−2.55	−4.31 ± 1.09
	V794 (C/S)	10	−5.33	−3.44	−4.55 ± 0.67
Matrix	V695 (F)	12	−12.24	−0.42	−6.05 ± 3.09
	V794 (C/S)	6	−18.76	−5.82	−15.95 ± 4.98

Although many factors could explain the high variability observed between analyses, we reject the hypothesis that the results are entirely a result of diagenesis. Mineral alteration has undoubtedly taken place to some extent, but we acknowledge that dietary, behavioral, ecological, and environmental factors can have likewise substantial effects on the isotopic signature of a given sample^45^. For example, significant differences between the δ^13^C and δ^18^O of the matrix and the teeth, as well as the δ^13^C of goniopholidid teeth and theropods in V794, show that the entire sample has not been isotopically homogenized by ground water replacement. In addition, the carnivorous theropods in this study have similar enriched δ^13^C values as seen in previous studies of dinosaurs from the Late Cretaceous^1^. Finally, comparisons with Suarez *et al*.’s^17^ phosphate isotope data set on the same microsite material (goniopholidid teeth from V794) show an offset of approximately + 4.5‰ between the phosphate and carbonate phase δ^18^O. These values are lower than the approximately + 7‰ mean average difference obtained in Late Cretaceous hadrosaur tooth enamel^46^, but nonetheless promising, given variation between sample and preparation in the two studies.

For herbivores, differences in δ^13^C values are commonly derived from the base of the food chain, reflecting contemporarily by differences in photosynthetic pathways between plant types (C3 vs C4 carbon fixation). The Mussentuchit ecosystem likely lacked appreciable C4 producers^47^ and thus the majority of δ^13^C differentiation at the base of the food chain would be caused by variation in the uptake and retention of CO_2._ In this system, the differences between microsites may represent varying degrees of canopy cover. Closed canopy forests, in general, tend to exhibit lower carbon isotope ratios due to the effects of plant respiration and decomposition near the forest floor^48^. Carbon in tooth carbonate is derived from ingested organics and modified through fractionation based on the consumer’s metabolic processes^1^. These differences are further exaggerated with each step up the food chain, as predators will often increase by approximately one per mil in relation to their prey^49^. δ^18^O in tooth carbonate is derived from ingested water and is variable based on the source of the water and the body temperature of the organism^1^.

These data indicate that the theropods, as a whole, did not differ substantially in bulk diet or ingested water, though given their significant difference in size and shape, the subtle isotopic variation between morphotypes are still worthy of discussion. δ^18^O showed no noticeable trend, indicating that all organisms (here theropods and goniopholidids) used similar water sources. This is a somewhat surprising result, since Suarez *et al*.^17^ found that theropods (mean δ^18^O = 18.3 ± 1.0‰) in V794 had a relatively depleted phosphate δ^18^O compared to goniopholidids (mean δ^18^O = 16.3 ± 1.0‰). Differences in the relative stability of the phosphate mineral and the size classes sampled (only presumed immature crocodilians) may explain the differences between the studies. δ^13^C had more interesting results, where goniopholidids were on average isotopically the most depleted. In modern environments δ^13^C is reflective of relative environmental cover and trophic level, meaning that these small crocodilians were likely eating trophically-low organisms and/or organisms living in a well-vegetated environment. Modern young crocodilians all transition through a similar ontogenetic dietary progression, with the youngest individuals relying heavily on invertebrates, which is gradually replaced by a diet of fish (e.g.^50^) followed by (depending on the species and environment) an additional switch to a diet including mammalian and reptilian components^51^. Isotopically, this is reflected in the tissues, as demonstrated by Radloff *et al*.^52^ in *Crocodylus niloticus*. Scute keratin samples from individuals showed at least two transitions in δ^13^C correlated with the length of the individual (SVL – length from tip of the snout to the end of the first scale row after the cloaca). The smallest animals hypothetically ate primarily invertebrates and showed a wide-ranging isotopic signature with a mean of approximately −21‰. This δ^13^C trend decreased linearly as the animal grew to an average of −25‰ at approximately 130 cm in SVL, likely reflecting an increasing level of fish in the diet of these individuals. Finally, at 240 cm SVL, the δ^13^C values dramatically increase to approximately −16‰, resulting from an increased diet of C4 consuming mammals with the onset of larger sizes.

If young goniopholidids were ecologically similar to living crocodilians, then we can assume that these depleted isotopic signals represent animals feeding on small, trophically-low, and possibly aquatic fauna (e.g. small fish and crustaceans). In V695, theropod morph 4 has a similar mean and range δ^13^C as the immature goniopholidids, implying a similar dietary type for both animals. In V794, morph 4 is more enriched than the goniopholidids, but still remains more depleted on average than morph 3 and morph 1, and further contains the most-depleted sample analyzed for any morphs (−6.4‰). Morph 2 shows the largest relative change between sites (from most enriched on average to least). Between the sites, the absolute differences are roughly equal for the goniopholidids (±0.10‰) and morph 2 (±0.11), but more substantial for morphs 3 (±1.37‰), 4 (±1.66‰), and 1 (±1.68‰). Ecologically, these differences can be explained by a shifting toward higher trophic foods for each of these morphs, a possible result of longer food chains in more aquatic environments. More interesting, however, is the observation that morph 2 is relatively depleted in environments in which it is poorly represented, possibly indicating a lack of dietary plasticity in this species and a reliance upon food sources more common in the floodplain setting. Though tantalizing as it may be to speculate on a behavioral basis for this disparity, caution will be taken here as not to over-interpret the isotopic results. Taken as a whole and given the high variability observed in these analyses, we interpret these results as suggestive, rather than indicative, of dietary niche partitioning between these theropods.

## Conclusions

Piscivory is hypothesized to have been a relatively common form of dietary partitioning in theropod dinosaurs. Many species of theropods have morphological features consistent with modern, fish-eating species; some to an absurd degree, such as the procumbent front teeth in the noasaurid *Masiakasaurus knopfleri*^53^. Evidence beyond morphological grounds is tentative, but supported for many of these species. For example, at least one specimen of the four-winged dromaeosaurid *Microraptor gui* preserves fish remains within its gut region^54^; however, other specimens preserve the remains of a mammal and a bird^55,56^, indicating that this species was a more generalist predator and not primarily feeding upon fish. Spinosaurid theropods show the morphological adaptations and geochemical signal of an aquatic predator^57^. Indeed, gut contents from the spinosaurid *Baryonyx walkeri* preserve acid-etched fish scales, but like the previous example of *Microraptor*, *B. walkeri* also preserves the remains of a juvenile iguanodontian^58^. Further, an embedded spinosaur tooth in a pterosaur vertebra indicates that this group would feed on other prey items besides fish^59^.

Like these examples, the species investigated here do not show obligate dietary or behavioral patterns for a single prey item or environment. Instead we see subtle, but nonetheless diagnostic patterns associated with differing lifestyles. *Richardoestesia* teeth (morph 4) are by far the most distinct, with a hypothesized preference (based on distribution among sites) for channel environments, and a variable isotopic signature consistent with a ^13^C-depleted diet. In contrast, the medium dromaeosaur (morph 2) is found more often in floodplain settings, but only varies slightly isotopically, depending on the environment in which it is found. Morph 1, the largest theropod, shows low abundance in both environments and has a geochemical signal consistent with an animal higher on the food chain. Last is morph 3, a small dromaeosaur, which is found in high abundances in all environments and maintains relatively diverse δ^13^C values, typical of a small-bodied opportunistic predator.

Morphs 1 and 2 have the lowest abundances near channel deposits. Although it is almost certain that these species would frequent any available water source at least temporarily, the data seemingly indicate that this was not a place of substantial tooth loss or, by inference, a preferred habitat. One possible explanation for this distribution is that these medium-sized theropods were competitively excluded from the near-water settings by large-bodied coelognathosuchian crocodilians. Some of these species potentially reached sizes over 5 m^20^, and their teeth are some of the most commonly encountered vertebrate fossils found in the MM^19^. Alternative hypotheses ranging from poor pedal traction near water to overgrown hunting terrain could also be viable explanations for this distribution.

**Figure 7:**
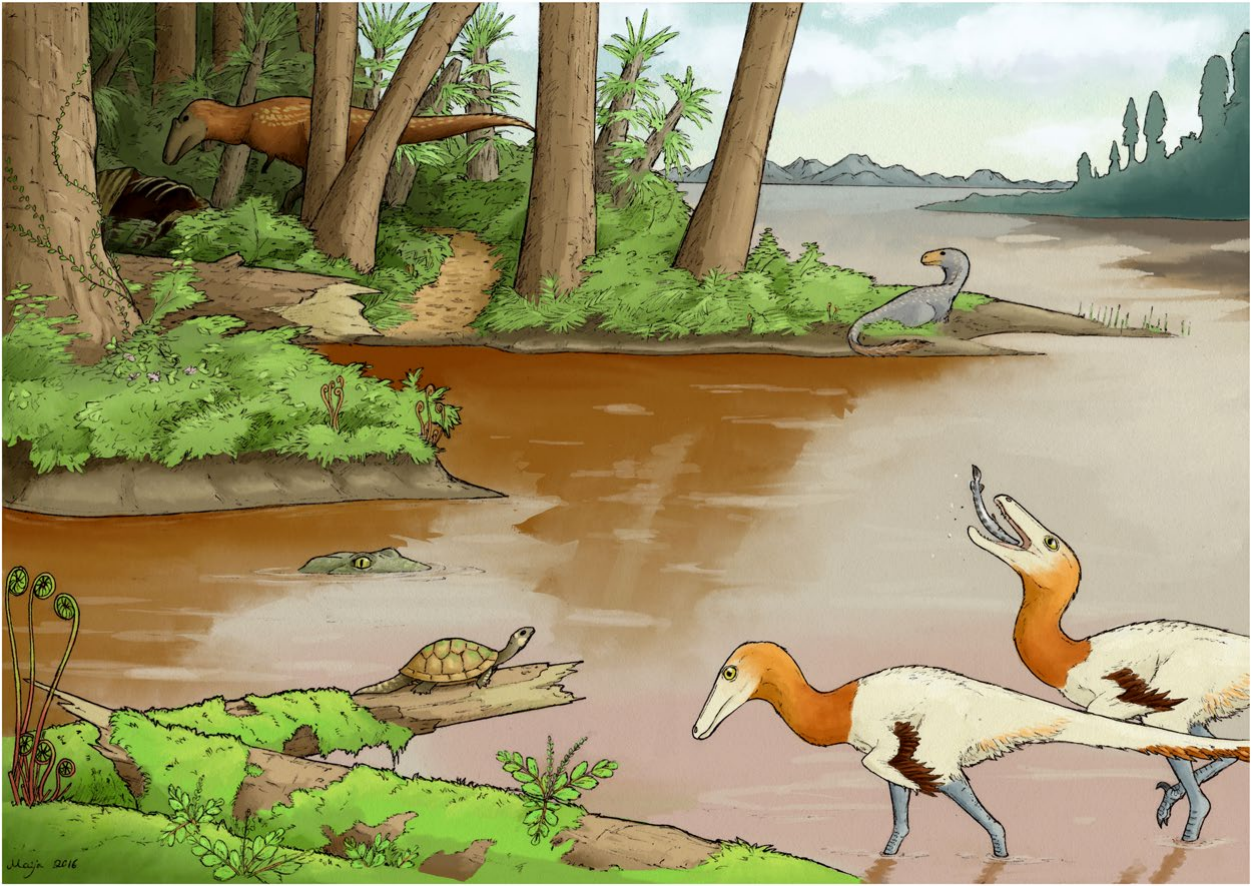
An artist**’**s reconstruction of V794. Image by Maija Karala.

In summary, we have shown here that it is possible to recover an ecological signal in theropod fossils without the need for direct evidence from preserved stomach content or feeding events. Through morphological, taphonomic, and geochemical proxies, we recognize that at least one species of MM theropod (morph 4 - *Richardoestesia* sp.) habitually lived and fed in aquatic environments, presumably specialized for a diet of small vertebrates (Fig. 7). Previous hypotheses for piscivory in this species are tentatively supported, though an obligate diet of fish or aquatic organisms can likely be dismissed. Individually, each line of investigation (morphology, depositional environment, and Carbon isotopes) is inconclusive; collectively, however, the results are consistent with niche partitioning within this medial Cretaceous theropod community.

## Methods

The first step of this analysis was to identify visually distinct tooth morphotypes from the bonebeds. Once morphs were recognized, specimens were identified and grouped using a Nikon SMZ-10A dissecting microscope. Incomplete specimens were not included in the analysis unless they could be assigned to one of the morphotypes with a high degree of confidence. Specimens were then measured, using hand calipers, for crown height, fore-aft basal length, basal width, and denticles per one mm (rounded to the nearest whole denticle) at the curve on both the posterior and anterior carinae^6^. In order to assess the validity of the provisional (subjectively-established) morphological groupings, measurements (with length, width, and height data Log-transformed to control for size) were analyzed with a discriminant analysis using PAST3^60^.

Next, to determine trophic ecology, we used a multi-tiered approach. First, tooth size was contrasted using a Kruskal-Wallis Test of tooth crown height for all complete specimens, to determine whether statistically significant size differences between tooth crown height exist between the morphs. Next, we ran a χ^2^-test to compare the distribution of morphotype between sites and to determine if any showed preference for a particular depositional setting, following Lyson and Longrich^61^. In order to test for taphonomic sorting bias, specimens for the most abundant tooth type (morph 3) and goniopholidid crocodilians were compared for crown height between two sites using a Mann—Whitney Test for Equal Medians of tooth crown height. Finally, we determined the stable isotope composition of carbonate associated with tooth enamel and/or dentin (δ^13^C and δ^18^O) to approximate the diet and habitat for each morph. Specimens for geochemical analysis were taken from incomplete, but morphologically distinguishable specimens for each morphotype, as well as small (<10 mm tall) goniopholidid teeth and matrix samples for comparison. Ideally, samples would be taken only from enamel (see^45^); however, many of the teeth were too small or had insufficient amounts of enamel for analysis alone. Dentin has more pore space than enamel and is more susceptible to groundwater alteration; however, given that these samples come from the same microsites (and thus the same diagenetic history) we assume that we are increasing type II error. By including this material, we acknowledge that the values obtained are the coarsest approximation for diet and habitat. Isotope samples were treated following a modified technique of Koch *et al*.^62^. Powdered specimens were first treated for one day using a 2% solution of NaOCl, washed five times with distilled water and then allowed to dry overnight, followed by a three-day treatment using 0.1 M acetic acid solution. Next, the samples were washed 10 times with distilled water and allowed to dry for three days.

The carbonate samples analyzed for their stable carbon and oxygen isotope compositions as follows. Approximately 200–300 µg of each carbonate was loaded into a 12 ml borosilicate exetainer vial (Labco 938 W) which were sealed with butyl rubber septa caps. The vials were then placed in a thermostated sample tray heated at 50 °C and flushed with ultra-high purity He (99.999%) using a ThermoGas Bench II equipped with a PAL auto sampler flushing needle for 360 seconds to remove the air. Then 0.4 ml of 100% phosphoric acid was manually injected into the vials with a syringe and the reaction was allowed to proceed at 50 °C for two hours. The vials were then sampled with the PAL measurement needle and the headspace CO_2_ was analyzed for δ ^13^C and δ^18^O using a Thermo Delta V Plus isotope ratio mass spectrometer. The carbon and oxygen isotopic compositions are expressed as^63^:

$${{\rm{\delta }}}^{{\rm{13}}}{{\rm{C}}}_{{\rm{VPDB}}}=[{\rm{R}}{({}^{{\rm{13}}}{\rm{C}}/{}^{{\rm{12}}}{\rm{C}})}_{{\rm{P}}}/{\rm{R}}{({}^{{\rm{13}}}{\rm{C}}/{}^{{\rm{12}}}{\rm{C}})}_{{\rm{VPDB}}}]-{\rm{1}}$$

and

$${{\rm{\delta }}}^{{\rm{18}}}{{\rm{O}}}_{{\rm{VPDB}}}=[{\rm{R}}{({}^{{\rm{18}}}{\rm{O}}/{}^{{\rm{16}}}{\rm{O}})}_{{\rm{P}}}/{\rm{R}}{({}^{{\rm{18}}}{\rm{O}}/{}^{16}{\rm{O}})}_{{\rm{VPDB}}}]-{\rm{1}}$$

where ${\rm{R}}{({}^{{\rm{13}}}{\rm{C}}/{}^{{\rm{12}}}{\rm{C}})}_{{\rm{P}}}={\rm{N}}{({}^{{\rm{13}}}{\rm{C}})}_{{\rm{P}}}/{\rm{N}}{({}^{{\rm{12}}}{\rm{C}})}_{{\rm{P}}}$ which is the ratio of the number of ^13^C and ^12^C atoms in sample P and equivalent parameters apply for VPDB and where R(^18^O/^16^O)_P_ = N(^18^O)_P_ ∕N(^16^O)_P_ which is the ratio of the number of ^18^O and ^16^O atoms in sample P and equivalent parameters apply for VPDB.

The δ^13^C values of the calcite samples are reported relative to VPDB on a scale normalized such that the δ^13^C of NBS18 is −5.01‰^64^. The δ^18^O_VPDB_ values of the calcite samples are reported on a scale normalized such that the δ^18^O of SLAP is −55.5‰ relative to VSMOW. On this δ^18^O_VPDB_ scale, the values of NBS 18 and NBS 19 are −23.01‰ and −2.2‰, respectively^65^. The oxygen isotope acid fractionation factor for calcite used for 50 °C is 1.00934^64^. The calcite δ^18^O_VSMOW-SLAP_ values were converted from the δ^18^O_VPDB_ values by using the IUPAC-recommended relation^64^:

$${{{\rm{\delta }}}^{{\rm{18}}}}_{\mathrm{OVSMOW} \mbox{-} \mathrm{SLAP}}={\rm{1.03092}}\,{{\rm{\delta }}}^{{\rm{18}}}{{\rm{O}}}_{{\rm{VPDB}}}+{\rm{30}}\mathrm{.92}{\rm{\textperthousand }}$$

The isotopic data were finally compared using the Mann-Whitney Pairwise test.

## Electronic supplementary material

Supplementary dataset

## Data Availability

Data are available through the supplementary data file.
